# Longitudinal and Noninvasive Intracellular Recordings of Spontaneous Electrophysiological Activity in Rat Primary Neurons on Planar MEA Electrodes

**DOI:** 10.1002/adma.202412697

**Published:** 2025-01-10

**Authors:** Rustamzhon Melikov, Giuseppina Iachetta, Marta d'Amora, Giovanni Melle, Silvia Conti, Francesco Tantussi, Michele Dipalo, Francesco De Angelis

**Affiliations:** ^1^ Italian Institute of Technology Genoa 16163 Italy; ^2^ Department of Biology University of Pisa Pisa 56127 Italy; ^3^ Foresee Biosystems srl Genoa 16121 Italy

**Keywords:** action potential, bioelectronics, intracellular recording, micro electrode arrays, optoacoustic poration

## Abstract

Presently, the in vitro recording of intracellular neuronal signals on microelectrode arrays (MEAs) requires complex 3D nanostructures or invasive and approaches such as electroporation. Here, it is shown that laser poration enables intracellular coupling on planar electrodes without damaging neurons or altering their spontaneous electrophysiological activity, allowing the process to be repeated multiple times on the same cells. This capability distinguishes laser‐based neuron poration from more invasive methods like electroporation, which typically serve as endpoint measurement for cells. It is demonstrated that planar MEA electrodes, when combined with laser cell optoporation and live cell staining, can record spontaneous intracellular signaling from primary neurons in vitro. This approach allows for the detection of attenuated signals resembling positive monophasic intracellular action potentials. Recordings after laser optoporation also reveal subthreshold signals such as post‐synaptic potentials that are essential for assessing neuronal network plasticity and connectivity. Moreover, the noninvasiveness of the process enables repeated intracellular recordings over multiple days from the same cells.

## Graphical abstract

An approach is presented to noninvasively porate neurons on planar MEA electrodes and enable the recording of intracellular‐like action potentials without interfering with the spontaneous activity of the network not affecting the neuronal health. In particular, it is shown that the method works on commercial MEA electrodes integrated on both high density and high‐throughput MEAs.

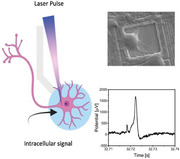

## 1 Introduction

In recent years, we have witnessed the impressive development of old and new methodologies for brain investigations. However, despite these great advances, the available methods still appear inadequate with respect to the enormous complexity of the human brain. For example, whereas brain capabilities are most likely emerging from large networks of neuronal populations, the available electrophysiological methods typically provide only an average observation of neuronal signaling limited to spatial and temporal scales, often fragmented. In this regard, significant results have been obtained by active and passive microelectrode arrays. These approaches enable investigations on the network level even though they typically access only the extracellular signals which are noisy and with poor information content. Great improvements have been achieved by exploiting engineered nano‐ and micro‐structures or 3D nanostructures^[^ ^1^, ^2^, ^3^, ^4^, ^5^, ^6^ ^]^ to access the intracellular compartment. However, very few successful approaches have been reported for neuronal cells.^[^ ^7^ ^]^ It's worth noticing that the practical implementation of 3D nanostructures is limited due to complex and expensive procedures that are not well‐suited for large‐scale production. A further disadvantage of current approaches for intracellular recording is the use of electroporation to access the intracellular compartment, as electroporation is highly invasive and may easily lead to cell detachment, perturbation or even cell death.^[^ ^7^ ^]^

In a previous investigation, we demonstrated the effectiveness of both 2D and 3D plasmonic nanostructured electrode to absorb and concentrate incident light radiation within their nanometric tips. These nanostructures act as transducers, converting light into the emission of hot electrons that generate nano shockwaves.^[^ ^8^ ^]^ This localized phenomenon, named optoacoustic poration, locally disrupts the plasma cell membrane of neurons, enabling the recording of intracellular neuronal action potentials on MEA.^[^ ^5^, ^9^ ^]^

Notably, we showed that such plasmonic metamaterials enabled intracellular recordings of high‐quality action potentials even on planar structures. However, the intracellular recording was limited to cardiac cells.^[^ ^10^ ^]^ The difficulties lie in the fact that, as we mentioned above, neurons are well known to be incredibly sensitive to any external stimulus. Hence membrane poration methods, including those based on electroporation, bring to cells silencing (interruption of firing), displacements (cells move away of the electrode), detaching (2D tissues disentanglement), tonic firing, or even death.

Here, we show that intracellular recording of spontaneous electrical activity can be achieved in rat primary neurons by exploiting laser optoacoustic poration of cell membrane. The method has a success rate of 88% with fluorophore staining when using commercial nanoporous (Pt HD MEA from 3Brain) and polymeric planar electrodes (PEDOT:CNT MEA from MCS). The poration attempt was considered unsuccessful if intracellular signal was not observed after three laser pulse shots. The high signal to noise ratio allows the observation of subthreshold neuronal signals on both high and low‐density MEAs. Importantly, we demonstrate that the method shows no evident side effects (low invasiveness). Hence, in opposition with electroporation or any other poration method, it allows the neurons to survive the process for further intracellular measurements or multiple consecutive poration events. The latter can be done even on the same cell/electrode for different days thus opening the way to longitudinal investigations without perturbation of the spontaneous activity or the need of evoking it. The technique exploits the optical and electrochemical performance of nanoporous metals and rough PEDOT:PSS surfaces with no need of 3D structures. To highlight the versatility of the approach and its potential impact on real‐world applications, we show results obtained on off‐the‐shelf commercial MEAs from two different suppliers.

In the following, we first describe the morphology and optical properties of the electrodes. Then, we structured the rest of the discussion into separate sections dedicated to the different employed MEA electrodes. In one case, we demonstrate optoporation of neurons on Pt HD‐MEAs, which are desirable for high‐resolution mapping of brain processes. In the second case, we show equivalent results on PEDOT:CNT MEAs that are ideal for higher throughput applications, as they can be produced in multiwell configurations up to 24/48/96 wells. Finally, we show that multiple consecutive poration can be done over the same cell/electrode thus enabling longitudinal investigations.

## 2 Result and Discussion

**Figure 1:**
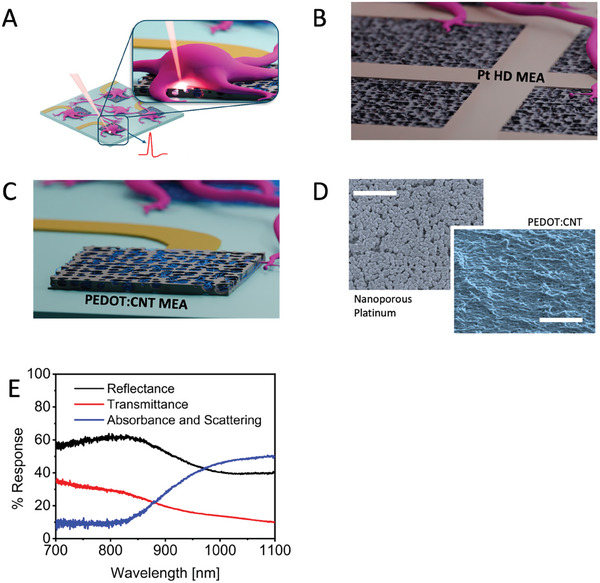
A) Schematic of MEA and a neuron sitting on MEA. B) Schematic of Pt HD MEA C) Schematic of PEDOT:CNT MEA D) SEM image of Pt HD MEA (scale bar – 1 µm) and PEDOT:CNT MEA (scale bar – 1 µm). E) Reflectance, transmittance, absorbance and scattering of individual electrodes with PEDOT:CNT MEA.

**Figure** **1** outlines the concept idea of laser poration on planar MEA electrodes and the morphological and optical properties of the materials under examination. In Figure 1A, we present a schematic representation of optoacoustic poration applied to neurons cultured on MEA. Briefly, the MEA with cultured neurons is placed on a microscope (upright or inverted) for applying focused laser radiation at the interface between MEA electrodes and cells (Figure 1A). We employed single‐well MEAs from two suppliers, Multi Channel Systems GmbH (MCS) and 3Brain AG. MCS‐MEAs consist of 60 electrodeposited PEDOT:CNT electrodes having a diameter of 30 or 10 µm with an inter‐electrode pitch of 200 µm (PEDOT:CNT MEA), whereas 3Brain MEAs consist of 4096 nanoporous platinum electrodes having a size of 18 µm and a pitch of 40 µm (Pt HDMEA) (Figure 1B,C). The platinum surface displays a porous structure characterized by significant roughness at the nanoscale, along with vacant nanogaps interspersed among the platinum protrusions (Figure 1D). Similarly, PEDOT:CNT electrodes have high roughness that enhances the neuron electrode interface (Figure 1D). PEDOT‐based electrodes gained interest in the past years due to their cost‐effective fabrication, reproducible impedance, and optimal biocompatibility that promotes tight neuron‐electrode coupling.^[^ ^11^, ^12^, ^13^ ^]^ The graph depicted in Figure 1E illustrates reflectance and transmittance data obtained via Fourier‐transform infrared spectroscopy of PEDOT:CNT electrodes on 30 µm MCS‐MEAs. In Figure 1E, we also plot the trend of absorbance and scattering derived from the measurements of transmittance and reflectance.

We can observe that PEDOT:CNT electrodes show high light absorption and scattering in the near‐infrared range from 900 to 1100 nm. In particular, the reflectance reaches a minimum of approximately 50% in the region between 1000 and 1100 nm, indicating that a large portion of the radiation is either absorbed or scattered at the interface between electrode and cell when the radiation is focused on the electrode surface. This feature is essential for achieving cell poration using low laser intensities, reducing thus possible photocytotoxicity effects on neurons.

Nanoporous Pt electrodes on high‐density MEAs (HD‐MEAs) show similar optical properties in the NIR range and are therefore ideal for optical coupling with laser radiation. An optical characterization of these electrodes may be found in the paper by Dipalo et al.,^[^ ^10^ ^]^ where they were used for poration and intracellular recording on cardiac cells.

### 2.1 Intracellular Recordings on Pt HD MEA

Active MEAs such as the Pt HD‐MEA from 3Brain are ideal devices for neuronal electrophysiology thanks to their high spatial resolution and large amount of recording units. These features enable the reconstruction of complex connections among thousands of neurons, providing higher data reliability and unique insights into the information processes of neuronal networks.

**Figure 2:**
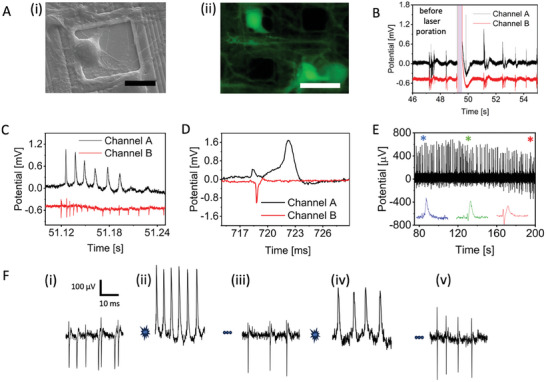
A) Images showing rat primary neurons cultured on nanoporous Pt HD MEA ((i) SEM image, scale bar – 10 µm; (ii) fluorescence image of live stained neurons, scale bar – 20 µm). B) Poration of rat primary neurons with power of 3.7 mW and pulse length 400 ms on a nanoporous Pt HD MEA (red line: neighboring electrode). C) Zoomed‐in view of intracellular action potentials within a burst. D) Zoomed view of a single intracellular action potential with observable subthreshold signal. E) Long‐term recording of the intracellular action potential. F) Multiple instances of poration and recovery on rat primary neurons.

We used primary hippocampal neurons obtained from rats for our optoporation experiments, as these cells represent a well‐established model with decades of published research.^[^ ^14^, ^15^, ^16^, ^17^, ^18^, ^19^, ^20^ ^]^ The neurons were cultured on the Pt HD‐MEA and maintained in culture for up to 21 days in vitro (DIV) until they displayed spontaneous electrogenic burst activity as expected from mature neuronal networks (see Figure S1, Supporting Information). The cell density was set to obtain a homogenous distribution of neurons on the device, avoiding the formation of clusters or multilayer cell structures (see **Figure** **2**A). This configuration favors the optical imaging of the cells and the focusing of the laser beam at the interface between neurons and electrodes. However, the visual identification of neuronal soma with brightfield microscopy remains challenging in the upright configuration on substrates with many features such as CMOS‐MEAs. It is worth noticing that the challenges we addressed here were twofold: the sensitivity of neurons and their smaller size compared to cardiomyocytes. Our previous works utilized laser raster scanning to porate entire cell cultures, but this approach proved suboptimal for neurons. In raster scanning, poration occurs at random locations, but neurons require precise poration at specific sites on the soma. Misplaced poration, for example on the side of the soma, can lead to very poor signals. In light of that, we opted for neuronal staining so to manually select the poration points. Looking ahead, this manual approach can be automated using image recognition software to identify soma positions and direct laser shots accordingly. We used a live cell staining protocol with NeuroFluor NeuO so that neurons would become fluorescent and could be unequivocally discriminated from astrocytes.^[^ ^21^ ^]^

Before applying laser optoporation, we record neuronal signals in the form of extracellular spikes, which exhibit a remarkably high signal‐to‐noise ratio with amplitudes ranging from approximately 100 to 1000 µVpp and the typical negative phase shape of extracellular field potentials. The cultures show spontaneous and synchronized activity as expected for neuronal networks after more than 21 DIVs, with an average mean firing rate (MFR) of 4 spikes s^−1^ (Figure 2B). These values are in line with results reported in the literature for neuronal cultures on CMOS‐MEA.^[^ ^22^ ^]^

To establish intracellular coupling, we first identify a neuron whose soma lays directly on the electrode, then we expose the electrode and the cell to an ultra‐fast‐pulsed 1064 nm laser (with a pulse train duration of 20–200 ms and average power ranging from 1 to 4 mW). The laser is focused on the interface between the neuron and the electrode, with an estimated spot size of 2 µm in diameter. Following laser excitation, we observe an immediate change of shape in the signals recorded from the stimulated electrode. After laser poration, signal shapes resemble intracellular APs, characterized by a complete positive phase followed by repolarization (Figure 2B, poration event at *t* = 49 s, Figure S6, Supporting Information). Therefore, Pt effectively functions as a meta‐electrode for plasmonic optoacoustic poration, enabling the delivery of reliable intracellular signals.

The average power required for optoacoustic poration of neurons on nanoporous platinum is comparable to that used with gold nanopillars.^[^ ^23^ ^]^ This significant outcome can be attributed to the morphology of Pt, which facilitates hot electron emission under light excitation even at lower electric fields.^[^ ^23^ ^]^

The intracellular neuronal action potentials induced by laser optoporation demonstrate synchronicity with neighboring electrodes, as depicted in Figure 2C (highlighted in red). This confirms that the poration event is not altering the spontaneous activity of the neuron nor its connections with the rest of the network. Indeed, in the example shown in Figure 2B, the MFR changes from 6.98 to 7.2 spike/s after the poration event, indicating very minor changes. When zooming in on one of the bursts, each individual intracellular AP can be visualized (Figure 2C,D). Upon further magnification for a single intracellular AP, we observe post‐synaptic potential alongside the single intracellular AP. It is evident that the membrane potential undergoes depolarization until reaching the threshold potential, resulting in the firing of an action potential from the neuron. Moreover, we calculated signal to noise ratio of up to 25 dB, which is sufficient to discriminate the shape of the action potential.

Remarkably, we could observe intracellular APs for several minutes after laser optoporation. Not only the neuronal cell does not alter its spontaneous electrophysiological activity after poration, but it also shows signs of recovery over time as the cellular membrane is able to reform and close the transient nanopores produced by laser poration. We can observe this phenomenon in Figure 2E, where we plot the continuous recordings of intracellular APs over a time window of 2 minutes. Initially, intracellular APs exhibit a typical shape of neuronal firing (blue trace inset*). As the neuronal membrane reforms, the signal amplitude decreases (green trace inset*). When the transient nanopores are almost completely closed, the signals acquire again the typical shape of extracellular field potentials (red trace inset *) (see Figure S9 (Supporting Information) for average intracellular coupling time).

As laser optoporation does not affect neuronal health, we also explored the possibility of repeating the poration process on the same neuron multiple times so that recordings of intracellular APs can be prolonged and repeated. We report an example of such an experiment in Figure 2F. Panel F(i) depicts extracellular neuronal spikes from an electrode on HD‐MEA before the application of laser optoporation with firing rate of 14.1 spike/s. In panel F(ii), we show signals from the same neuron after the first application of laser poration. The firing rate was observed as 13.3 spike/s and there was no drastic change in the firing rate of the neuron after optical poration. After a few minutes, signals come back to extracellular spikes as depicted in panel F(iii) (11.3 spike/s). At this point, we apply again laser optoporation and can obtain again an immediate change to the intracellular AP shape (panel F(iv)) (9.2 spike/s). The cell can then recover again by repairing the cellular membrane as shown in the recovery of extracellular spikes in panel F(v)(10.0 spike/s).

Neuronal intracellular APs induced by laser optoporation on HD‐MEA show amplitudes in the range of 0.4–2 mV. The relatively low amplitude of the signals compared to full action potentials is to be attributed to two main factors. On one hand, the transient nanopores in the cellular membranes are produced in a very small region defined by the laser‐focused spot, which is only 2 µm in diameter. As described in previous works, the rupture of the membrane is produced by localized shockwaves which occur under specific conditions. Within the area illuminated by the laser there are likely few spots which are able to produce nano‐shockwaves and, therefore, few small pores. This limits the area of intracellular coupling between the cell and the planar electrode of the HD‐MEA and then the signal intensity. On the other hand, it strongly reduces the perturbation of the neurons that continue their spontaneous activities.

Also, we notice that we cultured the neurons with relatively low density to avoid clustering. This way, the cells do not completely cover the MEA surface and thus leave the electrodes partially exposed to the cell medium, which defines the reference potential of the recordings. As a result, HD‐MEA electrodes intracellularly coupled with neurons are still affected by the cell medium, and the resulting APs are attenuated. This is also supported by measurements performed on HD‐MEA electrodes that pick up signals from more than one neuron. When laser optoporation is applied to a neuron on such an electrode, we observe burst activity with the presence of extracellular spikes as well as intracellular APs, coming respectively from the unperturbed neuron and the porated neuron (Figure S2, Supporting Information). These experimental data demonstrate that laser optoporation is an extremely localized phenomenon that does not affect neighboring cells. The data also highlight a unique feature of plasmonic optoporation on planar MEA electrodes, namely the possibility of using the same electrode for measuring and discriminating signals coming from different neurons with a much easier process of signal sorting, as the shape of extracellular and intracellular signals can be easily discriminated.

Despite the low amplitude, the high quality and high signal‐to‐noise ratio of the acquired intracellular APs enable the observation of subthreshold neuronal activity, which is a key element for characterizing neuronal networks and their connectivity.

Overall, these results demonstrate that laser optoporation can drastically improve the quality of neuronal signals acquired on high‐resolution Pt HD‐MEAs, enabling the detection of subthreshold potentials in addition to intracellular‐like action potential shapes. Moreover, the described poration process proves to be completely noninvasive for the primary neurons that do not show alterations in their spontaneous activity, detachment, or signs of apoptosis.

### 2.2 Intracellular Recordings on PEDOT:CNT MEA

MCS‐MEAs represent a widely adopted platform in electrophysiology laboratories due to their technical advantages, including superior extracellular recordings, versatility, and robustness. Typically, MCS‐MEAs use titanium nitride (TiN) electrodes as these provide low impedance and high robustness. However, the company MCS also commercializes MEAs with PEDOT:CNT electrodes, which consist of a composite material based on PEDOT and carbon nanotubes. PEDOT:CNT provides superior performance in terms of cell adhesion and cell stimulation thanks to high roughness and low and reproducible impedance. In the latter we show intracellular recordings on MCS‐MEAs facilitated by plasmonic optoacoustic poration on PEDOT:CNT electrodes with diameter of 30 µm.

**Figure 3:**
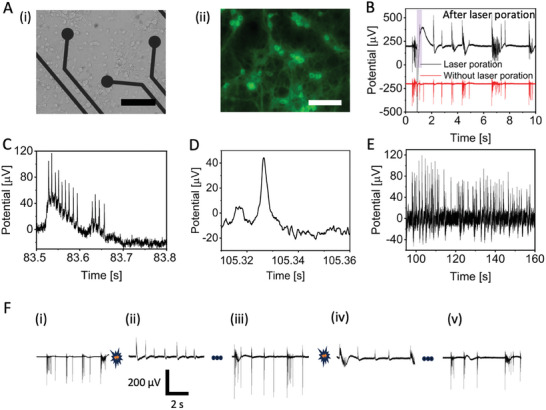
A) Images of rat primary neurons cultured on a PEDOT:CNT MEA (i) Brightfield image, scale bar – 100 µm; (ii) fluorescent image, scale bar – 100 µm). B) Poration of rat primary neurons with power of 6.5 mW and pulse length of 20 ms on a PEDOT:CNT MEA. C) Intracellular action potentials inside the burst. D) single intracellular action potential E) Extended recording of intracellular action potentials over time. F) Sequential poration events performed on rat primary neurons and subsequent recovery of extracellular action potentials.

For consistency with the previous results on Pt HD‐MEAs, we performed optoporation experiments on PEDOT:CNT MEAs using the same rat primary hippocampal neuronal model. A brightfield image of neurons cultured on PEDOT:CNT MEA is shown in **Figure** **3**A. The culture was homogenous and without clusters. As we used an upright microscope configuration to apply laser optoporation, we took advantage of fluorescent live cell staining to mark and visually identify the neurons (Figure 3A(ii)).

Neurons on PEDOT:CNT MEA form spontaneously active networks with synchronous bursting activity. At 21 DIVs, the neuronal cultures show an average MFR of 27.07 spike/s (see Figure S3, Supporting Information). Before laser optoporation, the amplitude of the recorded extracellular spikes ranges from approximately 100 to 500 µVpp (Figure 3B).

To establish intracellular coupling, we excite the interface between PEDOT:CNT MEA electrodes and neurons to an ultra‐fast‐pulsed 1064 nm laser (with a pulse train duration of 20–200 ms and average power ranging from 2 to 10 mW). As seen in the case of Pt HDMEAs, following laser excitation we observe an immediate change of shape of the recorded signals, moving from a predominantly negative phase to a positive signal resembling intracellular APs, characterized by a complete positive phase followed by repolarization (see Figure 3B). Thus, PEDOT:CNT effectively acts as a meta‐electrode for plasmonic optoacoustic poration, enabling the delivery of reliable intracellular signals.

The power required for optoacoustic poration on PEDOT:CNT ranges between 2 and 10 mW. These values are comparable to those used in the experiments on Pt HDMEA described in the previous section. This significant outcome can be attributed to the morphology of PEDOT:CNT, which facilitates hot electron emission under light excitation even at lower electric fields. As for the previous case, the synchronicity with neighboring neurons recorded from adjacent electrodes is preserved (Figure 3B, highlighted in red). It confirms that laser optoporation does not alter the spontaneous electrophysiological activity of the neuron nor its connections with the rest of the network.

Interestingly, in addition to the change of signal shape, after laser optoporation on PEDOT:CNT electrodes we also observe low‐frequency positive fluctuations in correspondence of neuronal bursts (Figure 3C). These potential fluctuations can be attributed to Giant Depolarizing Potentials (GDP), which are due to local electrochemical fields that induce membrane depolarization, resulting in action potential firing. The observation of GDPs on MEA after poration of neurons has been previously reported by Abbott et al.^[^ ^7^ ^]^ To be noted, we did not observe GDPs in extracellular recordings obtained before laser optoporation (Figure 3B).^[^ ^24^, ^25^ ^]^

Having achieved intracellular recordings on PEDOT:CNT electrodes with 30 µm diameter, we further explored the performance of optoacoustic poration on smaller PEDOT:CNT electrodes with 10 µm electrodes (Figure 1C,D). The area of these electrodes is much closer to the typical size of neuronal soma, resulting in a higher coverage of the electrode surface by the cells. In this scenario, the improved sealing of neurons on the smaller electrodes could result in intracellular signals with higher amplitudes. However, we did not observe statistically significant differences in intracellular signal amplitudes on 10 µm electrodes in respect to that obtained with 30 µm electrodes. This suggests that other mechanisms may contribute to the attenuation of the intracellular signals, such as the non‐optimal adhesion of the neurons on the electrode. The high seal resistance on small electrodes could bring more tangible improvements in the case of confluent cultures with higher cell density, which would fully cover and isolate the electrodes from the cell medium.^[^ ^7^ ^]^

Although we did not observe improvements in signal amplitude, the 10 µm electrodes did offer advantages in terms of sensitivity to small signals such as subthreshold potentials. Indeed, with 10 µm electrodes, we were able to detect excitatory postsynaptic potentials (EPSPs) as shown in Figure 3D. Moreover, the mean amplitude of the EPSP is in the quantal range^[^ ^26^ ^]^ (Figure S4, Supporting Information). PSPs were calculated by identifying baseline and SNR of the intracellular action potential. Then, we attributed small fluctuations less than half of the action potential to PSPs. We can observe that the membrane potential depolarizes until reaching the threshold potential, resulting in neuron firing followed by an undershoot. Furthermore, stable intracellular APs lasting more than 2 minutes were observed (Figure 3E) (see Figure S9, Supporting Information for average intracellular coupling time).

Additionally, multiple consecutive porations were made on the same electrode (Figure 3F) with the aim of testing invasiveness and the repeatability of the process. Panels F(i) and F(ii) show spontaneous neuronal activity before and after laser poration respectively. After a few minutes, we can observe in panel F(iii) that signals from the same electrode recover the typical extracellular shape, indicating full recovery of the cell membrane and successful closure of the membrane nanopores. We then apply a second laser pulse to induce again the opening of nanopores in the neuronal membrane. Successfully, we observe intracellular signals once again (Figure 3F and iv), and the subsequent recovery of the neuronal cell membrane as the signal comes back to the extracellular shape (Figure 3F and v).

**Figure 4:**
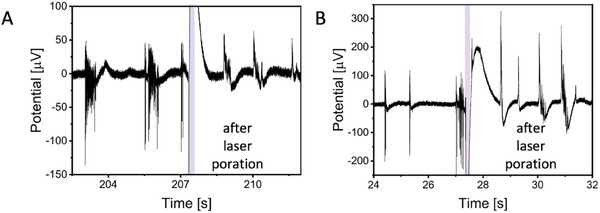
Poration and intracellular AP recordings of rat primary neurons at different DIVs on the same electrode within the same cell culture on PEDOT:CNT MEA. A) 20 DIV B) 21 DIV.

Remarkably, we were also able to apply laser optoporation and obtain intracellular AP recordings at different DIVs on the same electrode within the same cell culture, showing that optoporation may be repeated over days without affecting the neuronal network (**Figure** **4**). The artifact created by the laser pulse lingers 1.07 s more after the end of 20 ms laser pulse (Figure S8, Supporting Information). This opens the way to longitudinal investigations of electrical activity at intracellular level, a goal not yet reached.

Overall, we successfully applied laser optoacoustic poration and recorded attenuated intracellular action potentials (APs) over 6 cell cultures, 21 PEDOT:CNT MEAs, involving over 200 cell porations. The selection of electrodes for optoacoustic poration was limited to those in direct contact with neuronal cell bodies and recording extracellular field potentials with significant amplitudes (>100 µV), indicating tight coupling with the cells that is crucial for successful poration. The amplitude of the intracellular signals was in the range of 100–300 µV, with peaks reaching up to 500 µV under optimal coupling conditions. The duration of intracellular signals was approximately 1–2 ms, reflecting the typical values of intracellular APs in neuronal cells. Also in the case of PEDOT:CNT electrodes, we attribute the relatively low amplitude of intracellular APs to reasons similar to those described earlier for Pt HDMEAs. The used PEDOT:CNT electrodes had a diameter of 30 µm, which is much larger than the typical size of neuronal soma. Hence, a large portion of the electrode is exposed to the cell medium while intracellular potentials are picked up in a very small region corresponding to the laser spot size (approx. 2 µm diameter).

The success rate of optoacoustic poration on PEDOT:CNT MEAs is 88% with most of the laser pulse trains resulting in intracellular recordings, demonstrating the reproducibility of the process. Specifically, in 3 cultures using fluorophore staining, we successfully completed 22 porations out of 25 attempts, resulting in an 88% success rate. Additionally, we tested poration without staining in a total of 441 cells and success rate dropped to 42%. Overall, these findings highlight that optoacoustic poration is well‐suited for reliable intracellular recordings in large cultures using commercial MEAs with PEDOT:CNT electrodes.

### 2.3 Electrical Activity of Neurons Before Poration and Recovered Neurons after Poration

**Figure 5:**
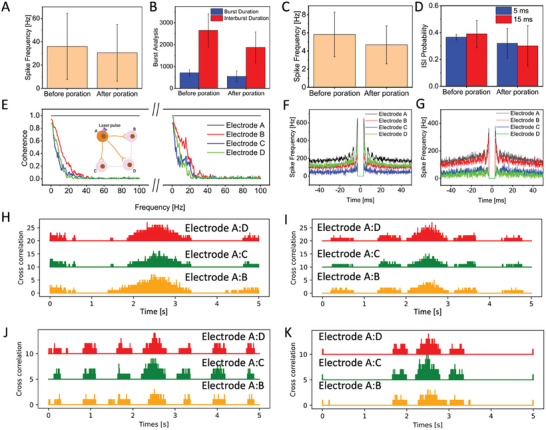
A) Average spike frequency before poration and recovered neurons after poration for cultured neurons on PEDOT:CNT MEAs (*N* = 3, p value = 0.92). B) Burst and Interburst duration before poration and recovered neurons after poration for cultured neurons on PEDOT:CNT MEAs (*N* = 3, p value = 0.93(blue), p value = 0.98(red)). C) Average spike frequency before poration and recovered neurons after poration for cultured neurons on Pt HDMEAs (*N* = 3, p value = 0.95). D) ISI probability before poration and recovered neurons after poration for cultured neurons on Pt HDMEAs (*N* = 3, p value = 0.86(blue), p value = 0.94(red)). E) Coherence of neighboring electrodes to porated electrode before and recovered neurons after poration on cultured neuron on PEDOT:CNT MEA.(In the inset, four interconnected neurons A–D, and neuron A is porated). F–G) Autocorrelation of porated and neighboring electrodes before after (G) poration on PEDOT:CNT MEA. H) Cross correlation of electrode A with its neighbors before optoporation on PEDOT:CNT MEA. I) Cross correlation of electrode A with its neighbors after optoporation on PEDOT:CNT MEA. H) Cross correlation of electrode A with its neighbors before optoporation on Pt HD MEA. I) Cross correlation of electrode A with its neighbors after optoporation on Pt HD MEA.

One of the primary benefits of optical poration of neurons is its non‐invasive nature. To support this claim, we analyzed in detail the network activity of neuronal cultures before and after optoporation to evaluate whether the application of laser pulses could change or influence the network‐level communication. We start by analyzing the spiking and bursting activity of neurons on MCS MEAs before and after optoporation on a neuron. Data shown in **Figure** **5**A demonstrate that neuronal activity on PEDOT:CNT MEA remains unchanged after the application of optoporation, with a p‐value of 0.92. Furthermore, burst duration and interburst duration exhibit no significant variations after optoporation, with p‐values of 0.93 (red) and 0.98 (blue), respectively. Similarly, neurons cultured on Pt HDMEAs show no significant change in activity after poration, as shown in Figure 5C with a p‐value of 0.95. The interspike interval (ISI) probability at 5 ms and 15 ms also remains consistent, with p‐values of 0.86 (red) and 0.94 (blue). Overall, these datasets support the fact that network activity in terms of mean firing rate and burst frequency do not change during and after laser optoporation.

However, a non‐invasive access to the intracellular neuronal compartment should also leave unchanged the connection circuits within the network, especially between the porated neurons and the remaining cells. This allows for correlating the subthreshold signals acquired from the porated neuron with the plasticity of the whole culture. Thus, we next evaluated the electrophysiological activity of porated neurons in respect to connected electrodes. To do so, we calculated coherence and autocorrelation of electrodes adjacent to the porated sites. The data show no significant change compared to before poration conditions (Figure 5E–G). Additionally, Figure S5 (Supporting Information) illustrates that the burst behavior of the neuronal culture is preserved after poration (Figure S5A–D, Supporting Information). Similarly, the maintenance of network activity is evident in cross‐correlograms (Figure 5H–K). These findings underscore the advantages of the laser poration method over traditional electroporation techniques in the literature.^[^ ^7^ ^]^

## 3 Conclusion

In this study, we demonstrated that planar MEA electrodes combined with laser cell optoporation and live cell staining enable the recording of intracellular signaling from primary neurons in vitro. In addition to a clear change of shape of the recorded signals after poration, which resemble positive monophasic intracellular APs, our data present also distinctive subthreshold signals such as post‐synaptic potentials that are fundamental for assessing plasticity and connectivity of neuronal networks.

A major key advantage of the approach is the noninvasiveness of laser poration, which enables intracellular coupling without damaging the neurons nor affecting their spontaneous electrophysiological activity, providing the possibility to repeat the process multiple times on the same cells. Such a feature distinguishes laser‐based neuron poration from other methods like electroporation that are extremely invasive and do not offer the possibility of repeated or prolonged investigations. We show this feature both in short‐term recordings, where we porate the same neurons within minutes and in long‐term recordings, where we porate neurons on the same electrode on different days.

The non‐invasiveness of laser poration also plays a key role in the type of activity that can be recorded on MEA. Indeed, after laser poration the neurons preserve their spontaneous electrical activity and this allows for analyzing the physiological network activity without interfering nor needing for external stimuli, such as electrical eliciting of action potentials or current clamping.

Lastly, we highlight that the results presented in this work have been obtained using two different types of commercial MEAs already well‐established on the market, without post‐processing or modifications. Hence, the approach may be replicated and applied on a large scale in real‐world neuroscience applications.

## 4 Experimental Section

### Rat Primary Hippocampal Culture on Commercial PEDOT:CNT MEAs and Pt HD‐MEAs

Primary rat hippocampal neurons purchased from Lonza (Walkersville, MD, United States; Catalog No. R‐HI‐501). These neurons were derived from embryonic brain tissue (E18 and E19) and were ready for immediate culture with no need for further ethical approval. Neurons were cultured in Primary Neuron Growth Medium (PNGM, Lonza, Walkersville, MD, United States) supplemented with 2 mM L‐glutamine, 50 µg mL^−1^ gentamicin, 37 ng mL^−1^ amphotericin, and 2% of Neural Serum Factor 1 (NSF) (Lonza PNGM Singlequots Growth Supplements), in a humidified incubator under an atmosphere of 5% CO_2_ at 37 °C.

The PEDOT:CNT MEAs underwent UV sterilization for 30 minutes. Following sterilization, a 30 µg mL^−1^ poly‐D‐lysine (Sigma‐Aldrich, St. Louis, MO, USA) and 2 µg mL^−1^ laminin (Sigma‐Aldrich, St. Louis, MO, USA) solution in Phosphate Buffer Saline (PBS, pH = 7.4, Thermo Fisher Scientific, Inc., Waltham, MA) was dropped on the culture surface area of the devices and left for 1 h at room temperature, enhancing neurons adhesion and proliferation on the MEAs. After coating, the chips were washed three times with sterile water (Sigma‐Aldrich, St. Louis, MO, USA) and air‐dried inside the hood, before cell seeding.

Pt HD‐MEAs were sterilized in 70% ethanol for 20 minutes, followed by three washes with sterile water, and dried under a sterile laminar flow hood. Subsequently, the Pt HD‐MEAs were pre‐conditioned by overnight incubation at 37 °C, 5% CO_2_, and 95% humidity with PNGM. The following day, the PNGM was aspirated, and the Pt HD‐MEAs were coated with a solution containing 30 µg mL^−1^ poly‐D‐lysine and 2 µg mL^−1^ laminin diluted in Phosphate Buffer Saline (PBS), then incubated for 4 hours at 37 °C, 5% CO_2_. Afterward, the Pt HD‐MEAs were washed four times with sterile water and dried overnight under sterile conditions before cell seeding.

Primary rat hippocampal neurons were seeded on the Pt HD‐MEAs and PEDOT:CNT MEAs (2 00 000 cells per device) and incubated for 4 hours until neurons settled. Next, part of the medium was carefully removed, and fresh medium was added to the well. Cell cultures were maintained for 3/4 weeks according to the cell supplier's protocol with the first medium change on day 5, replacing 50% of the medium with fresh medium, followed by regular replacements every 3–4 days. Electrophysiological recordings were performed 21 days after plating.

### NeuroFluor NeuO Staining

Primary rat hippocampal neurons were cultured on the devices for 21 up to 28 days. To visualize the neurons on the microelectrodes, primary rat hippocampal neurons were stained with NeuroFluor NeuO (STEMCELL Technologies, Vancouver, BC, Canada), a membrane‐permeable fluorescent probe for the detection of live neurons, before the measurements. A final concentration of 0.25 µM NeuroFluor NeuO was mixed directly into the culture media covering the device and incubated for 1 h in a humidified incubator at 37 °C with 5% CO_2_. Immediately after incubation, cells were measured.

### Electrophysiology Recording

Recordings from PEDOT:CNT MEA were performed at 37 °C outside the incubator by an MCS acquisition system, which could record 60 channels at a 25 kHz sampling rate. Recordings from pt HD‐MEA were performed at 37 °C outside the incubator by a 3Brain acquisition system, which could record 4096 channels at an 18 kHz sampling rate. All data sets were analyzed by Python custom code.

### Laser Optoacoustic Poration

Initially, a 5‐minute period of undisturbed extracellular activity was recorded to characterize the culture at filter intervals ranging from 100 Hz to 3500 Hz. Subsequently, laser pulse trains were employed on the surface of either MCS MEAs or 3Brain HDMEAs to induce poration of rat primary hippocampal cells. For laser poration, the first harmonic (λ = 1064 nm) of a Nd:YAG (neodymium:yttrium–aluminum–garnet) solid‐state laser, specifically the Plecter Duo model from Coherent, was utilized as the radiation source for the optoacoustic poration experiments, delivering an average power of approximately 4 mW after passing through the objective. The laser was integrated into a modified upright microscope (Nikon Eclipse FN1) capable of accommodating the acquisition system directly on the microscope stage. During the experiments, a 20X objective (NA = 1, working distance = 4 mm) was employed to observe the cells on the devices and to focus the near‐infrared (NIR) laser used for poration.

### Scanning Electron Microscopy (SEM)

Rat hippocampal neurons cultured on Pt HD‐MEA devices were fixed with a solution of 2% glutaraldehyde in Na cacodylate buffer (0.1 M) for 2 h at room temperature, washed three times with the same buffer, post‐fixed in 0.1% osmium tetroxide for 1 h and then rinsed twice with deionized water. Subsequently, the samples were dehydrated with a graded series of ethanol in water solutions (from 30% to 100%), followed by incubation in ethanol:hexamethyldisilazane (HMDS) and then in 100% HMDS. Finally, the samples were dried overnight in the air and coated with a 10 nm gold layer. Imaging analysis was performed using a dual beam Helios Nanolab600 by ThermoFisher.

### Statistical Analysis

Data from PEDOT:CNT MEA and Pt HD MEA was not pre‐processed. Brainwave 4 software was used from 3Brain to calculate spike frequency and ISI probability from Pt HD MEAs (*n* = 3). Multi Channel Systems Analyzer was used from MCS to calculate spike frequency and burst parameters (*n* = 3). Mean and standard deviation was calculated for the spike frequency, ISI probability, burst parameters (*n* > 3). Null‐hypothesis test was used to identify difference between before and after poration for parameters such as ISI probability, burst duration, interburst duration and spike frequency. Custom python code was used to calculate cross correlation from both Pt HD MEA and PEDOT:CNT MEA samples. NeuroExplorer software was used to calculate coherence and autocorrelation of neurons before and after the poration.

## Conflict of Interest

The authors declare no conflict of interest.

## Supporting information



Supporting Information

## Data Availability

The data that support the findings of this study are available from the corresponding author upon reasonable request.

## References

[1] T. Berthing , S. Bonde , K. R. Rostgaard , M. H. Madsen , C. B. Sørensen , J. Nygård , K. L. Martinez , Nanotechnology. 2012, 23, 415102. 23010859 10.1088/0957-4484/23/41/415102

[2] R. Liu , J. Lee , Y. Tchoe , D. Pre , A. M. Bourhis , A. D'Antonio‐Chronowska , G. Robin , S. H. Lee , Y. G. Ro , R. Vatsyayan , K. J. Tonsfeldt , L. A. Hossain , M. L. Phipps , J. Yoo , J. Nogan , J. S. Martinez , K. A. Frazer , A. G. Bang , S. A. Dayeh , Adv. Funct. Mater. 2022, 32, 2108378. 35603230 10.1002/adfm.202108378 PMC9122115

[3] A. Koklu , R. Atmaramani , A. Hammack , A. Beskok , J. J. Pancrazio , B. E. Gnade , B. J. Black , Nanotechnology. 2019, 30, 235501. 30776783 10.1088/1361-6528/ab07cd

[4] C. Xie , Z. Lin , L. Hanson , Y. Cui , B. Cui , Nat. Nanotechnol. 2012, 7, 185. 22327876 10.1038/nnano.2012.8 PMC3356686

[5] M. Dipalo , H. Amin , L. Lovato , F. Moia , V. Caprettini , G. C. Messina , F. Tantussi , L. Berdondini , F. De Angelis , Nano Lett. 2017, 17, 3932. 28534411 10.1021/acs.nanolett.7b01523 PMC5520104

[6] H. Amin , M. Dipalo , F. De Angelis , L. Berdondini , ACS Appl. Mater. Interfaces. 2018, 10, 15207. 29620843 10.1021/acsami.8b00387 PMC5934727

[7] J. Abbott , T. Ye , K. Krenek , R. S. Gertner , S. Ban , Y. Kim , L. Qin , W. Wu , H. Park , D. Ham , Nat. Biomed. Eng. 2019, 4, 232. 31548592 10.1038/s41551-019-0455-7 PMC7035150

[8] M. Dipalo , G. C. Messina , H. Amin , R. La Rocca , V. Shalabaeva , A. Simi , A. Maccione , P. Zilio , L. Berdondini , F. De Angelis , Nanoscale. 2015, 7, 3703. 25640283 10.1039/c4nr05578k

[9] P. Zilio , M. Dipalo , F. Tantussi , G. C. Messina , F. de Angelis , Light Sci. Appl. 2017, 6, e17002. 30167264 10.1038/lsa.2017.2 PMC6062236

[10] M. Dipalo , G. Melle , L. Lovato , A. Jacassi , F. Santoro , V. Caprettini , A. Schirato , A. Alabastri , D. Garoli , G. Bruno , F. Tantussi , F. De Angelis , Nat. Nanotechnol. 2018, 13, 965. 30104618 10.1038/s41565-018-0222-z

[11] E. He , S. Xu , Y. Dai , Y. Wang , G. Xiao , J. Xie , S. Xu , P. Fan , F. Mo , M. Wang , Y. Song , H. Yin , Y. Li , Y. Wang , X. Cai , ACS Sens. 2021, 6, 3377. 34410704 10.1021/acssensors.1c01241

[12] R. Samba , T. Herrmann , G. Zeck , J. Neural Eng. 2015, 12, 016014. 25588201 10.1088/1741-2560/12/1/016014

[13] R. Gerwig , K. Fuchsberger , B. Schroeppel , G. S. Link , G. Heusel , U. Kraushaar , W. Schuhmann , A. Stett , M. Stelzle , Front Neuroeng. 2012, 5, 8. 22586394 10.3389/fneng.2012.00008 PMC3343311

[14] D. Soscia , A. Belle , N. Fischer , H. Enright , A. Sales , J. Osburn , W. Benett , E. Mukerjee , K. Kulp , S. Pannu , E. Wheeler , PLoS One. 2017, 12, 0188146. 10.1371/journal.pone.0188146 PMC5697820 29161298

[15] G. Bruno , G. Melle , A. Barbaglia , G. Iachetta , R. Melikov , M. Perrone , M. Dipalo , F. De Angelis , Adv. Sci. 2021, 8, 2100627. 10.1002/advs.202100627 PMC8564419 34486241

[16] G. Bruno , N. Colistra , G. Melle , A. Cerea , A. Hubarevich , L. Deleye , F. De Angelis , M. Dipalo , Front. Bioeng. Biotechnol. 2020, 8, 626. 32656200 10.3389/fbioe.2020.00626 PMC7325920

[17] M. Niedringhaus , X. Chen , R. Dzakpasu , PLoS One. 2015, 10, 0129324. 10.1371/journal.pone.0129324 PMC4466488 26070215

[18] J. Fan , G. Thalody , J. Kwagh , E. Burnett , H. Shi , G. Lewen , S.‐J. Chen , P. Levesque , Toxicology. 2019, 55, 93. 10.1016/j.tiv.2018.12.001 30528373

[19] K. M. LaBarbera , C. Limegrover , C. Rehak , R. Yurko , N. J. Izzo , N. Knezovich , E. Watto , L. Waybright , S. M. Catalano , J. Neurosci. Methods. 2021, 358, 109180. 33836174 10.1016/j.jneumeth.2021.109180 PMC8217273

[20] H. Amin , T. Nieus , D. Lonardoni , A. Maccione , L. Berdondini , Sci. Rep. 2017, 7, 2460. 28550283 10.1038/s41598-017-02635-x PMC5446416

[21] J. C. Er , C. Leong , C. L. Teoh , Q. Yuan , P. Merchant , M. Dunn , D. Sulzer , D. Sames , A. Bhinge , D. Kim , S. Kim , M. Yoon , L. W. Stanton , S. H. Je , S. Yun , Y. Chang , Angew. Chem., Int. Ed. 2015, 54, 2442. 10.1002/anie.201408614 25565332

[22] N. Lama , A. Hargreaves , B. Stevens , T. McGinnity , presented at 2018 International Joint Conference on Neural Networks (IJCNN) , IEEE, New York 2018, pp. 1–8.

[23] A. Naldoni , U. Guler , Z. Wang , M. Marelli , F. Malara , X. Meng , L. V. Besteiro , A. O. Govorov , A. V. Kildishev , A. Boltasseva , V. M. Shalaev , Adv. Opt. Mater. 2017, 5, 1601031.

[24] A. Lombardi , P. Jedlicka , H. J. Luhmann , W. Kilb , Front Cell Neurosci. 2018, 12, 420. 30515078 10.3389/fncel.2018.00420 PMC6255825

[25] S. T. Sipilä , K. Huttu , I. Soltesz , J. Voipio , K. Kaila , The J. Neurosci. 2005, 25, 5280. 15930375 10.1523/JNEUROSCI.0378-05.2005 PMC6725004

[26] N. R. Hardingham , J. C. A. Read , A. J. Trevelyan , J. C. Nelson , J. J. B. Jack , N. J. Bannister , J. Neurosci. 2010, 30, 1441. 20107071 10.1523/JNEUROSCI.3244-09.2010 PMC2825095

