# Supplementary material for: Longitudinal and Noninvasive Intracellular Recordings of Spontaneous Electrophysiological Activity in Rat Primary Neurons on Planar MEA Electrodes
Source: Adv Mater. 2025 Jan 10;37(8):2412697. doi: 10.1002/adma.202412697 (PMC11854871; doi:10.1002/adma.202412697)
Supplement: Supplementary file 1 — Supporting Information [file ADMA-37-2412697-s001.docx]

Supporting Information

**Longitudinal and noninvasive intracellular recordings of spontaneous electrophysiological activity in rat primary neurons on planar MEA electrodes**

Rustamzhon Melikov, Giuseppina Iachetta, Marta d’Amora, Giovanni Melle, Silvia Conti, Francesco Tantussi, Michele Dipalo, Francesco De Angelis ^*^

**Activity of Neurons cultured on nanoporous Pt HDMEAs**


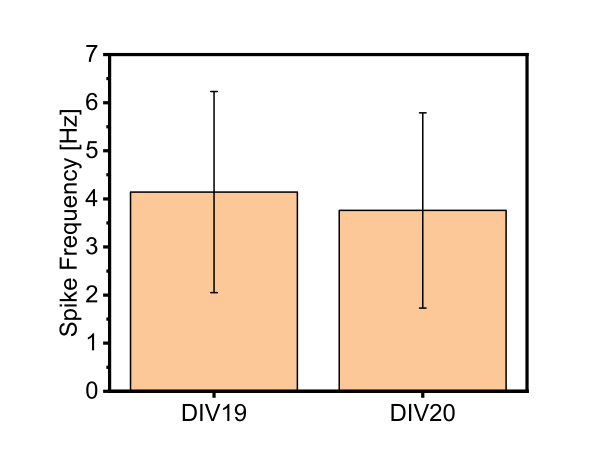


*Figure S1. Spike frequency at various DIV for neurons cultured on nanoporous Pt HDMEAs (N=3)*

**Simultaneous recording of both extracellular field potentials and intracellular action potentials**

When two neurons are active on a single electrode, as illustrated in the schematic and image in Figure S2A, the extracellular signal is recorded simultaneously from both neurons (Figure S2B). Upon targeting one of the neurons with laser optoporation, it becomes possible to detect an intracellular signal from the porated neuron while still measuring the extracellular signal from the unperturbed neuron (Figure S2C). Once the membrane nanopores close, only the extracellular signals from both neurons are observed again.


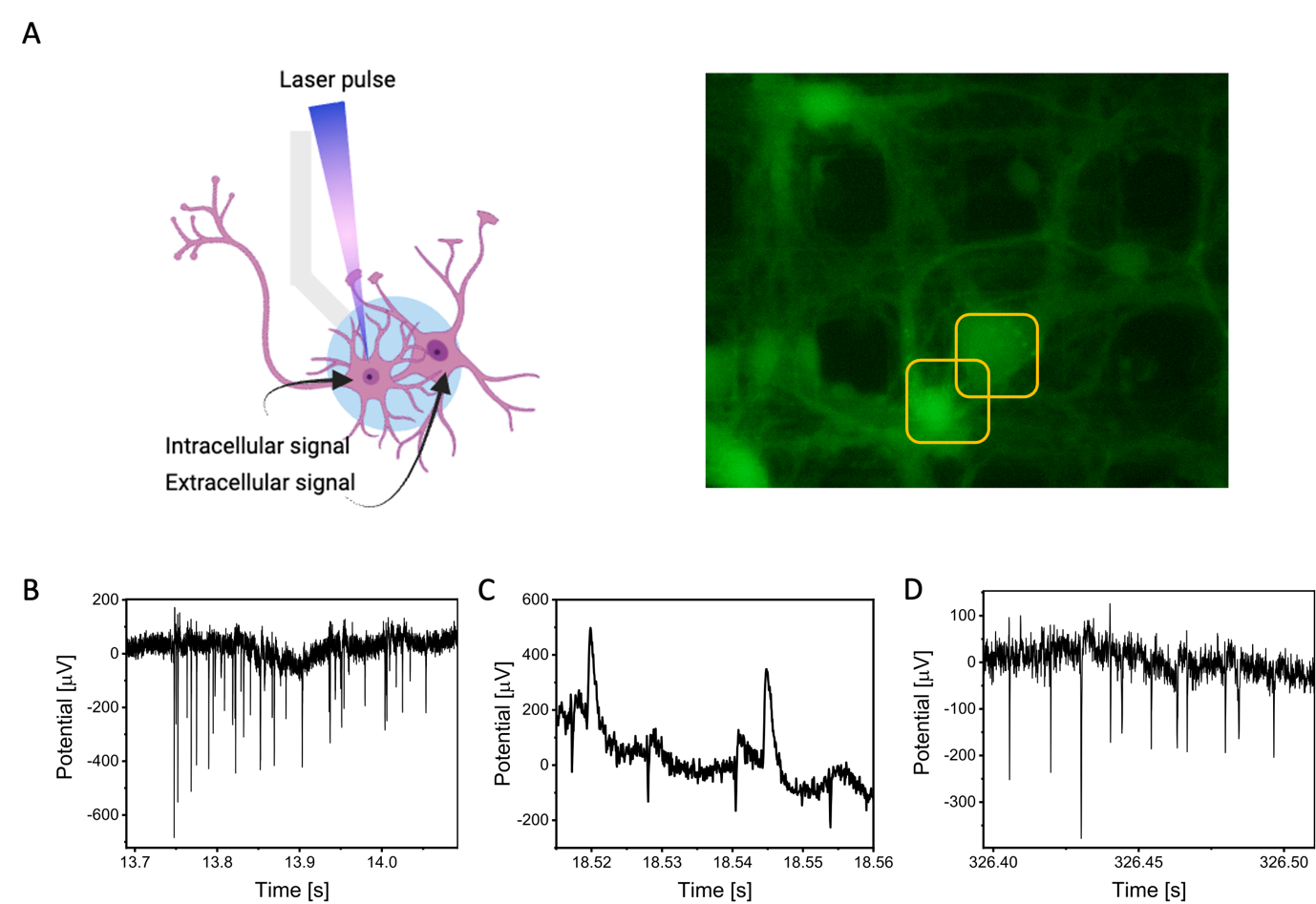


*Figure S2. Simultaneous Intracellular and Extracellular action potential recording. A) Schematic and image of two neurons on a single electrode (yellow rectangles highlight neuronal soma ) B) Extracellular signal before poration C) Mixture of intracellular and Extracellular signals D) Extracellular signal when pore is closed*

**Activity of Neurons cultured on nanoporous PEDOT:CNT MEAs**


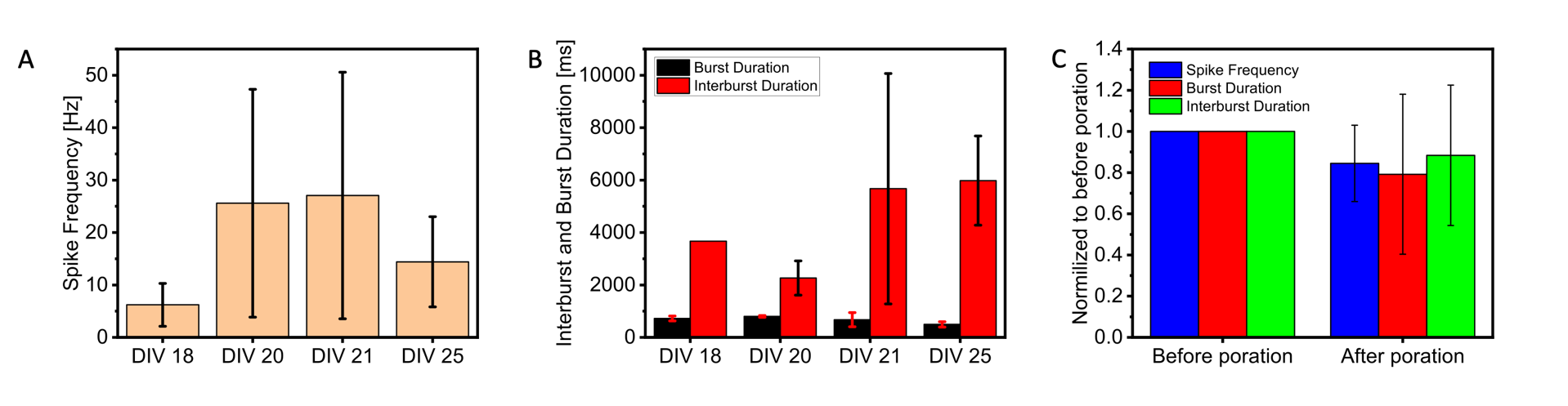


*Figure S3. A) Spike frequency at various DIV for cultured neuron on PEDOT:CNT MEA.(N=3) B) Interburst and burst duration at various DIV for cultured neuron on PEDOT:CNT MEA (N=3)*

**Average amplitude of EPSP signals**


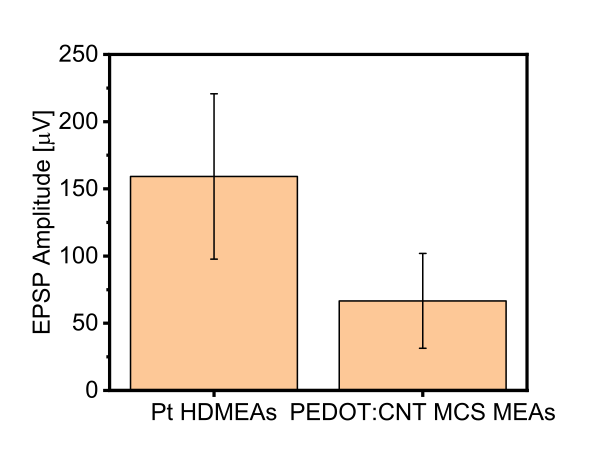


*Figure S4. Amplitude of EPSP signals observed after optoporation on Pt HDMEAs and PEDOT:CNT MEA.*

PSPs were calculated by identifying baseline and SNR of the intracellular action potential. Afterwards small fluctuations less than half of the action potential are potentially PSPs.

**Retaining burst behavior after optoporation attempt**


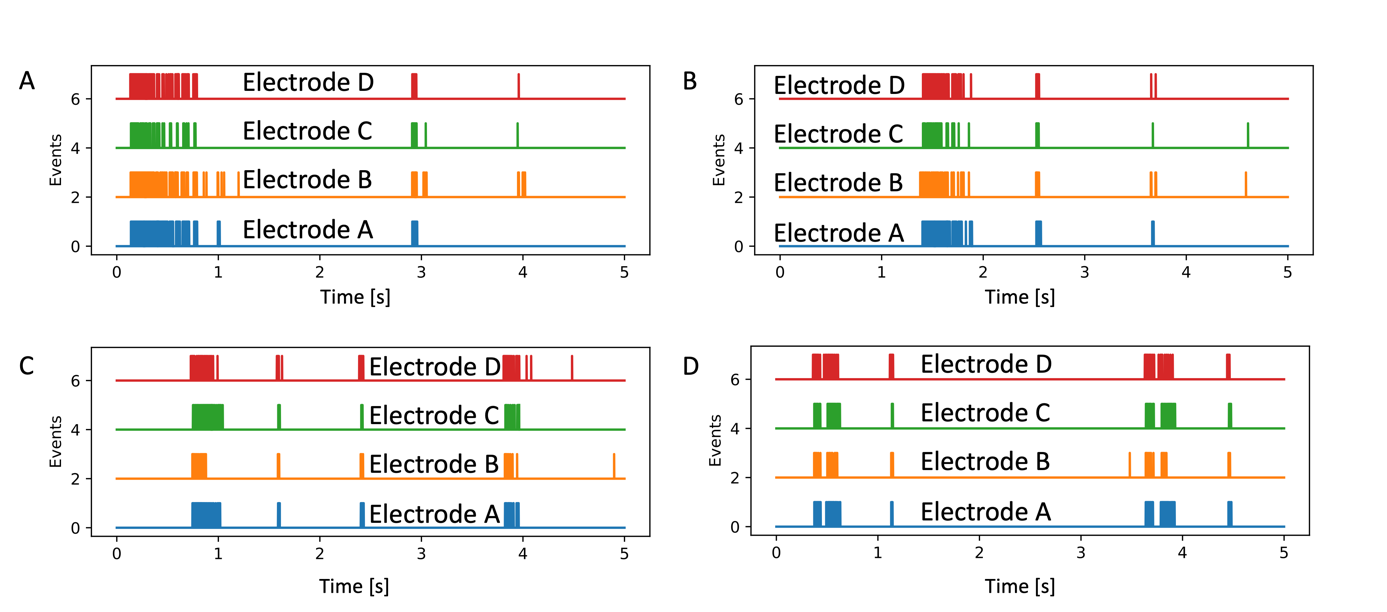


*Figure S5. A) Spike events before poration on the electrodes A and its neighbors on PEDOT:CNT MEA. B) Spike events after poration on the electrodes A and its neighbors on PEDOT:CNT MEA. C) Spike events before poration on the electrodes A and its neighbors on Pt HD MEA. D) Spike events after poration on the electrodes A and its neighbors on Pt HD MEA.*

**Electrical signal from primary neuron before and after poration**


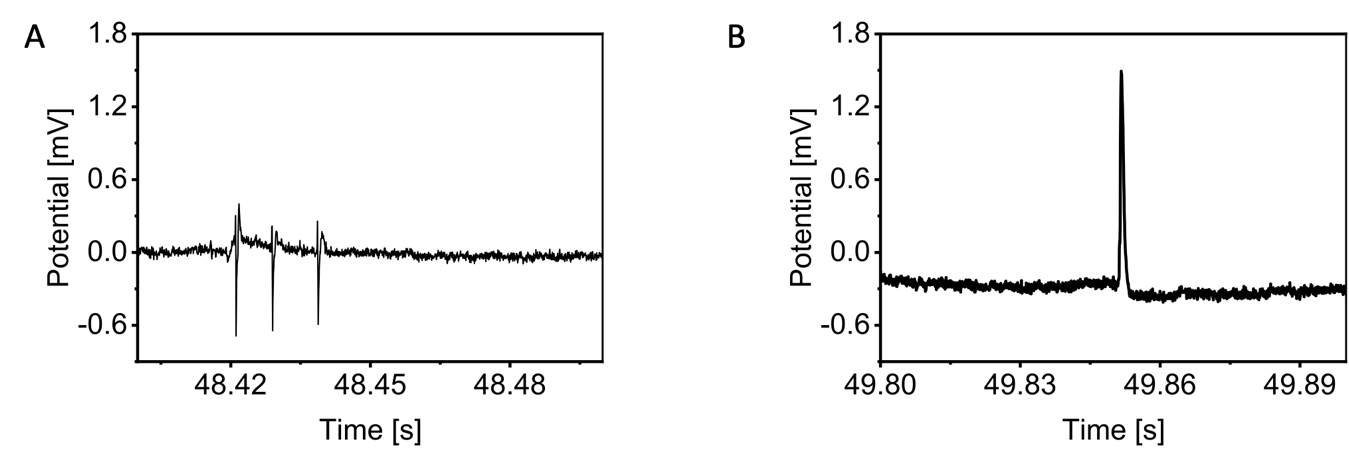


*Figure S6. A) Extracellular spike before poration attempt on pt HD MEA. B) Intracellular action potential after poration attempt on pt HD MEA*

**Electrical signal from primary neuron before and after recovery from 2^nd^ poration**


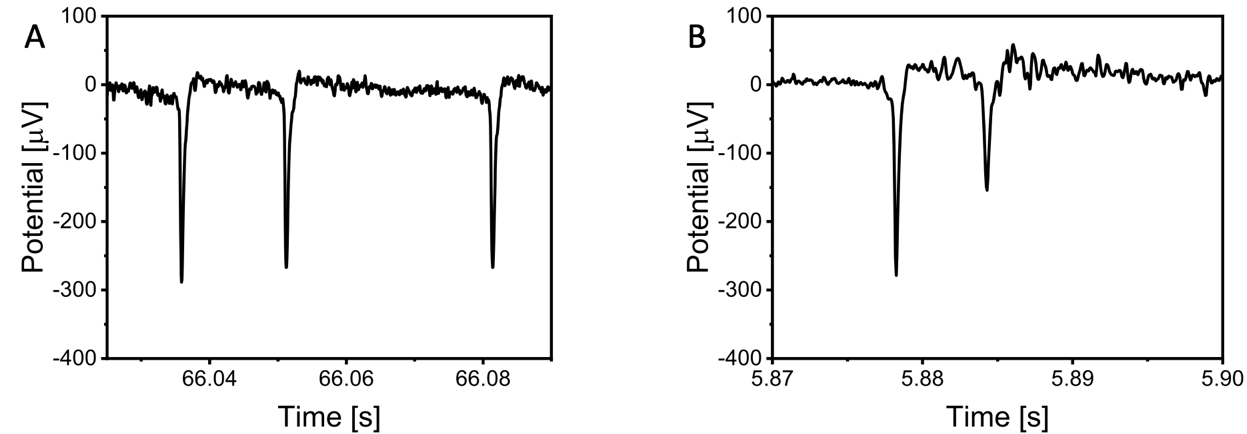


*Figure S7. A) Extracellular spikes before poration. B) Extracellular spikes after full recovery from double poration.*

**Response of MEA from 20 ms laser shot**

*
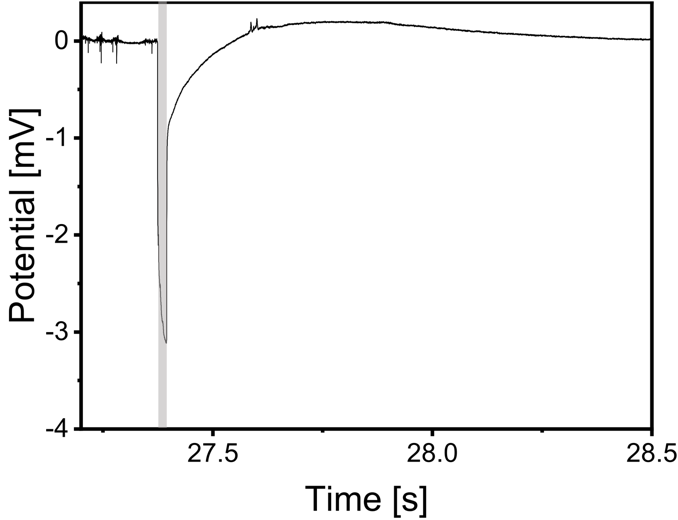
*

*Figure S8. Artifact created after applying 20 ms laser pulse (gray box). 1.07 s time is needed until recording stabilize under steady state (light gray box). (The figure zooms in figure 4B)*

**Intracellular coupling time**


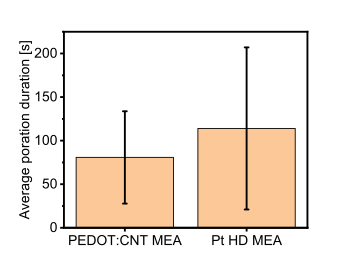


*Figure S9. Average poration duration of neurons on PEDOT:CNT MEA (n = 16) and Pt HD MEA (n=8)*
